# Fish scale rich in functional compounds and peptides: A potential nutraceutical to overcome undernutrition

**DOI:** 10.3389/fnut.2022.1072370

**Published:** 2022-12-09

**Authors:** Netty Salindeho, Jeffrie F. Mokolensang, Lusia Manu, Nurpudji Astuti Taslim, Fahrul Nurkolis, William Ben Gunawan, Muhammad Yusuf, Nelly Mayulu, Apollinaire Tsopmo

**Affiliations:** ^1^Fishery Products Technology Study Program, Faculty of Fisheries and Marine Sciences, Sam Ratulangi University, Manado, Indonesia; ^2^Aquaculture Study Program, Faculty of Fisheries and Marine Sciences, Sam Ratulangi University, Manado, Indonesia; ^3^Faculty of Fisheries and Marine Sciences, Sam Ratulangi University, Manado, Indonesia; ^4^Clinical Nutrition, Faculty of Medicine, Hasanuddin University, Makassar, Indonesia; ^5^Biological Sciences, State Islamic University of Sunan Kalijaga (UIN Sunan Kalijaga), Yogyakarta, Indonesia; ^6^Nutrition Science Department, Faculty of Medicine, Diponegoro University, Semarang, Indonesia; ^7^Medical Programme, Faculty of Medicine, Universitas Brawijaya, Malang, Indonesia; ^8^Faculty of Medicine, Sam Ratulangi University, Manado, Indonesia; ^9^Department of Chemistry, Carleton University, Ottawa, ON, Canada

**Keywords:** fish scale, bioactive peptides, nutraceutical, stunting, growth, undernutrition, bioactive compounds, supplementation

## Introduction

Child undernutrition remains an important global health problem. Undernutrition increases the susceptibility to illness and fatality and is related to 45% of child deaths (1). Undernourished children also have severe short-term (e.g., delayed cognitive development), medium-term (e.g., lower school achievement), and long-term implications (e.g., lower earnings and higher probability of adult non-communicable chronic diseases) (2). Child undernutrition is often a consequence of inadequate intake of vitamins and minerals such as vitamin A, iron, iodine, and zinc but also poor quality or insufficient proteins (3, 4). The manifestations are low weight for height (or wasting) and low height for age (or stunting). Furthermore, the incidence of stunting is greatly influenced by early life undernutrition since growth faltering frequently starts while a child is still in the womb and lasts for at least the first 2 years after birth (5, 6). In addition to these determinants, the availability of health services is important (7). The rates of stunting or chronic protein energy malnutrition are increasing in certain parts of the world. One of the proposed solutions is producing food or supplements rich in nutrition from the conversion of food by-products high in good-quality proteins.

Fish is a healthy food with a high nutritional value which makes it extremely important for the human food chain (8). Mass quantities of fish waste are produced annually during fish processing, where fish waste, including fish scales, is discarded (9). To date, fish waste is partly used for the production of fishmeal, fertilizers, and fish oil with low profitability or utilized as a raw material for direct feeding in aquaculture, while the rest are thrown away (10). Fish scales are frequently regarded as abandoned waste from the aquaculture industry, including fish canning, fileting, salting, and smoking processes (11). An estimated 7.2–12 million tons of fish waste are thrown away globally each year, with the 5 most utilized species being *Oreochromis niloticus, Sardinella brasiliensis, Pogonias cromis, Labeo rohita*, and *Leporinus elongatus* (11). The fish scale yields various functional applications originating from its valuable components such as hydroxyapatite, collagen, and chitin (10, 12). Furthermore, the collagen in fish scales can be utilized into bioactive peptides with various health benefits (13, 14). Many efforts and research are being carried out to exploit the potential of the fish scale, starting from the potential in the fields of nutrition and food to medicine (15, 16). However, the development of functional food from the fish scale to contribute to nutritional problem solutions is currently underdeveloped.

Fish-derived peptides exhibit various biological activities such as an angiotensin-I-converting enzyme (ACE) inhibitory activity, antioxidant, antimicrobial as well as anticancer activity, and immunostimulant activity (17). Peptides, in addition to their nutritional characteristics as sources of amino acids, are known to also have beneficial health effects, as they can present the ability to interact directly with human metabolism routes, acting as health promoters and in the mitigation of the aging process (18). Previous studies have identified many types of bioactive peptides derived from the fish scale. Four types of bioactive peptides from the sea bream (*Sparus aurata*) scale have been showing antihypertensive activity with various efficacies. Other bioactive peptides from different fish scales yielded antioxidant activities (10, 19). Regulation of oxidative stress and immunity plays an important role in the growth and physiological metabolism. Eventually, it could lead to the prevention of malnutrition, especially undernutrition conditions including stunting. A preclinical study by Sabrina et al. (20) showed that bioactive peptides could improve nutritional status biomarkers such as serum protein, hemoglobin, and IGF-1 levels. Stunting is a condition in which a child has a below-average height, which is two standard deviations lower than their age on the standard growth chart (21, 22). With its abundance in protein and bioactive peptides, fish scales showed interesting potential as a nutraceutical that could act to fulfill the unmet needs of the stunting population.

Therefore, this article aims to interpret the latest findings about the potential application of fish scales as a functional food that has functional compounds and peptides, which may have the potential to overcome undernutrition as a nutraceutical.

## Fish scale

Fish scales are composed of type I collagen and hydroxyapatite (16). The identification of the fish scale's major components revealed that moisture and protein share the majority of fish scale weight. Maktoof et al. (9) analyzed the scales of *Cyprinus carpio* fish, finding that between 22.1 and 23.9% of the scales' weight consists of protein with a low lipid and carbohydrate content. The proportion of protein tends to increase in association with the increase in weight and length of the fish (9). Due to its nutritional value, especially its high protein, some researchers were able to develop nutritional food and meals from fish scales (15).

Fish scales are the source of many valuable products. Fish scales consist of a type I collagen multilayer with orderly orientation, adequate mechanical strength, transparency, and good biocompatibility (23). Fish scales collagen gained advantages because it is considered a safer collagen source compared to other animal-derived scaffolds due to the absence of zoonotic infections and religious issues (16). Alongside collagen, gelatin is also a component of interest in fish scales. Gelatin belongs to a class of protein fractions derived from collagen by thermal hydrolysis which involves breaking hydrogen bonds between polypeptide chains of collagen molecules. Due to its characteristics, gelatin has the most significant application in the food industry field, pharmaceutical, and cosmetics industries (24). Gelatin supplementation can enhance joint and bone health (25). Fish-based gelatin also encourages tissue regeneration, raising bone marrow density and offering an alternate benefit for patients with osteoporosis (26).

**Figure 1:**
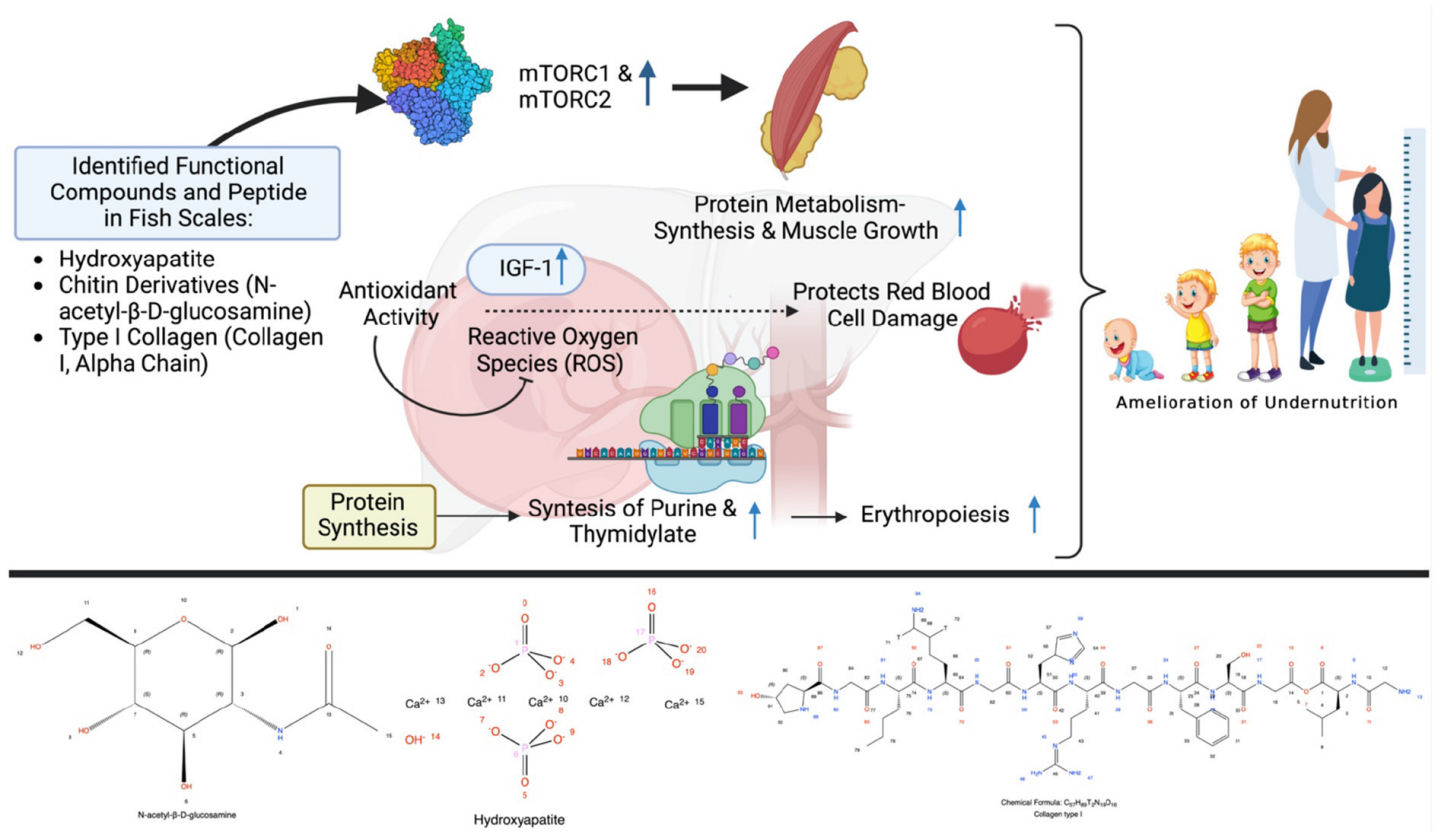
Possible scheme to alleviate stunting *via* the modulation of the metabolism by fish scale peptide supplementation. Created with BioRender.com premium license by Fahrul Nurkolis.

Aside from collagen, the fish scale also contains hydroxyapatite with various utilization values. Hydroxyapatite is the hydroxylated representative of phosphate minerals known as apatites [Ca_10_(PO_4_)_6_(OH)_2_]. Hydroxyapatite from fish scales has emerged as an alternative to substitute synthetic and bovine hydroxyapatite, due to the similarity of chemical properties that simple and inexpensive methods can achieve (Figure 1). Results from studies have shown that hydroxyapatite from fish scales demonstrated no cytotoxicity, increased mineralization *in vitro*, and tolerable biocompatibility in murine models (27). Hydroxyapatite constituent from fish scale was also developed as a calcium-binding peptide which promotes calcium cellular uptake (28). Those pieces of evidence suggested a significant role of hydroxyapatite in bone metabolism.

Chitin can also be found in fish scales. Chitin is a very attractive item owing to its biological properties and therapeutic feature *via* antibacterial and antifungal activities. Chitin is a long-chain odorless or tasteless amino polysaccharide of white or off-white color in its pure state, composed of *N*-acetyl-β-D-glucosamine units and monomers (Figure 1). The utilization of chitin derivatives is numerous, ranging from medical, pharmaceutical, food, and cosmetic industries, to nutraceuticals, bioremediation, gene therapy, and cosmetics (10). Chitin has many beneficial properties as an antioxidant, prebiotic, dietary fiber, and hypocholesterolemic agent (29). Incorporating chitin into a protein-based meal was also shown to improve growth, increase fatty acid production, and modulate gut microbiota (30).

## Fish scale supports growth and prevents malnutrition through various mechanisms

Food that are rich in protein show many health benefits which are influenced by the presence of bioactive peptides (31). The antioxidative, anti-inflammatory, anticancer, antimicrobial, immunomodulatory, and antihypertensive properties of bioactive peptides derived from dietary proteins are only a few of their important roles in the living body (17). Fish scale, which is a potential source of bioactive peptides, can be utilized to synthesize chitin and chitosan, which have antioxidant, antimicrobial, and antiviral properties (32, 33). These properties may contribute to the incidence of growth retardation since it involves immune dysfunction, antioxidant, and metabolic (hormonal) system (34). Antioxidant was shown to enhance the activity of insulin-like growth factor-1 (IGF-1; Figure 1) and its receptors (35), while growth hormones also reduce oxidative stress (36). An improvement in immunity will result in a good cellular metabolism through the activation of rapamycin (mTORC1 and mTORC2; Figure 1) which promote protein synthesis, glycolysis, mitochondrial functions, and lipid synthesis (37). Bioactive peptides also upregulate calcium uptake, which is associated with healthy bone growth (38). Collagen peptides from the fish scale also showed immunomodulatory activity by protecting cells from cytotoxicity and inflammation (39, 40). Collagen peptides made from fish scales contain a unique amino acid composition with a high concentration of proline, hydroxyproline, and glycine (41). Due to its ability to control cellular redox equilibrium, proline, a non-essential amino acid, plays an important role in protein structure or function and the regulation of illnesses through extensive metabolic networks (42). Collagen contains 57% of the total amino acids, mostly glycine, proline, and hydroxyproline, which is necessary to preserve the strength and regular structure of connective tissue, including bones, skin, cartilage, and blood vessels (43).

The antimicrobial activity derived from the fish scale may also play a role in preventing malnutrition, which is supported by a systematic and meta-analysis study that found that antibiotics – which treated infections and might modulate intestinal microbiota – promoted growth in children (44). Diarrhea, water supply, sanitation, and hygiene practices were significantly associated with the incidence of malnutrition (45). Preventing infection and diarrhea through the use of antimicrobial agents against Shigella, Vibrio, Salmonella, Campylobacter, and many others is genuinely recommended (46). A considerable amount of micronutrients, such as calcium, iron, magnesium, and phosphorus, were identified in fish scales (47). Calcium and magnesium had a significant contribution to bone and muscle health (48). Next to that, multiple micronutrient supplementations had shown good results by improving growth and reducing the risk of anemia in infants (49). Overall, this strategy may give a significant contribution to preventing anemia (a risk factor for stunting) in teenage girls or pregnant mothers (50) while also potentially resolving the dual-occurrence of anemia and stunting in children (51). Hemoglobin levels were positively correlated with growth hormone levels [e.g., insulin-like growth factor I (IGF-1)] which emphasized the role of hemoglobin in preventing growth retardation (52, 53). These facts highlight the fish scale as a wonderful source of both collagen and bioactive peptides which is rich in amino acid and micronutrients, supports growth, and prevents malnutrition through various mechanisms (Figure 1).

## Nutraceutical products and developments based on fish scale bioactive peptides

The processing and utilization of fish scales into a food product of health value (nutraceuticals) are a challenge for researchers. This opinion article attempts to interpret the latest findings about the potential application of fish scales as a functional food that has the potential to overcome undernutrition. However, we also aim to stimulate researchers in the exploration of bioactive peptides derived from fish scales. Therefore, there is a need for further research that focuses on this research topic. Unutilized fish scales may affect the realization of Sustainable Development Goals Number 14 (Life Below Water) since their waste can cause environmental pollution. Therefore, fish scales can be developed as functional food products through various technologies and methods, which may also reduce fish scale waste. Isolation, encapsulation, nanotechnology, and possibly fermentation are some of the alternative methods that can be used to achieve the purpose of utilizing fish skin bioactive peptides. More interestingly, fish scales have collagen composed of bioactive peptides. Supplementation of food products containing bioactive peptides in rats was shown to improve nutritional status biomarkers such as serum protein, hemoglobin, and IGF-1 levels (20). Collagen contained in fish scales will undergo a hydrolysis reaction to produce gelatin. Fish scale gelatin is a class of biopolymers containing abundant and potential bioactive amino acids and peptides, which can be utilized in savory products such as fish scale crispy (15), cookies (54), and protein hydrolysate (28). The natural characteristics of fish gelatin indicate that this fish scale gelatin product can be used as an ingredient in making jelly or agar-agar, both types of food are favored by children. This will be an added value in the intervention of malnourished children or will lead to stunting. Therefore, in addition, to being able to overcome environmental problems, the use of fish scales can also overcome nutritional problems.

## Conclusion

As explained previously, there is the potential for processing fish scales into a functional food product rich in bioactive peptides, which can not only overcome environmental problems, but this can also overcome nutritional problems, especially to overcome undernutrition (Figure 1). Natural processes in the body are modulated almost exclusively by the interaction of certain amino acid sequences, either as peptides or as subsections of proteins or polypeptides. In connection with growth, proteins and peptides are involved in the modulation of cell proliferation, cell migration, inflammation, metabolism (hormonal), and protein synthesis and regulation. Research on the therapeutic peptide or bioactive analogs of specific interactive sequences derived from fish scales has opened the door to a diverse new field of pharmaceutical ingredients and functional foods for the food industry. These facts form the basis that fish scales have the potential to be a source of collagen and bioactive peptides rich in amino acids and micronutrients, support growth, and prevent malnutrition through various mechanisms. It is suitable to be applied in nutritional interventions in children with stunting. Further clinical trials related to these benefits are expected to be conducted by many researchers.

## Author contributions

All authors listed have made a substantial, direct, and intellectual contribution to the work and approved it for publication.
